# Article processing charges for open access publication—the situation for research intensive universities in the USA and Canada

**DOI:** 10.7717/peerj.2264

**Published:** 2016-07-21

**Authors:** David Solomon, Bo-Christer Björk

**Affiliations:** 1 Internal Medicine/Office of Medical Education Research and Development, Michigan State University, E Lansing, MI, United States; 2 Information Systems Science, Hanken School of Economics, Helsinki, Finland

**Keywords:** APC charges, APC charges open access

## Abstract

**Background.** Open access (OA) publishing via article processing charges (APCs) is growing as an alternative to subscription publishing. The Pay It Forward (PIF) Project is exploring the feasibility of transitioning from paying subscriptions to funding APCs for faculty at research intensive universities. Estimating of the cost of APCs for the journals authors at research intensive universities tend to publish is essential for the PIF project and similar initiatives. This paper presents our research into this question.

**Methods.** We identified APC prices for publications by authors at the 4 research intensive United States (US) and Canadian universities involved in the study. We also obtained APC payment records from several Western European universities and funding agencies. Both data sets were merged with Web of Science (WoS) metadata. We calculated the average APCs for articles and proceedings in 13 discipline categories published by researchers at research intensive universities. We also identified 41 journals published by traditionally subscription publishers which have recently converted to APC funded OA and recorded the APCs they charge.

**Results.** We identified 7,629 payment records from the 4 European APC payment databases and 14,356 OA articles authored by PIF partner university faculty for which we had listed APC prices. APCs for full OA journals published by PIF authors averaged 1,775 USD; full OA journal APCs paid by Western European funders averaged 1,865 USD; hybrid APCs paid by Western European funders averaged 2,887 USD. The APC for converted journals published by major subscription publishers averaged 1,825 USD. APC funded OA is concentrated in the life and basic sciences. APCs funded articles in the social sciences and humanities are often multidisciplinary and published in journals such as PLOS ONE that largely publish in the life sciences.

**Conclusions.** Full OA journal APCs average a little under 2,000 USD while hybrid articles average about 3,000 USD for publications by researchers at research intensive universities. There is a lack of information on discipline differences in APCs due to the concentration of APC funded publications in a few fields and the multidisciplinary nature of research.

## Introduction

Since the launch of the first Open Access (OA) journals funded by Article Processing Charges (APC) around 2000, APC funded OA publication has grown rapidly. By 2010 the number of articles published in APC funded OA journals indexed in Scopus surpassed the number of articles published in OA journals funded by other means (Solomon, Laakso & Björk, 2013). There also is evidence that APC funded OA articles are continuing to grow exponentially. Between 2010 and 2012, the number of APC funded OA articles published by 7 major OA publishers more than doubled from 41,974 to 87,021 (Neylon, 2013). Along with publishers that only publish APC funded OA journals, large, traditionally subscription publishers, are rapidly increasing the number of OA journals they publish. For example, between August 2013 and June 2016 Elsevier increased the number of APC funded OA journals they publish from 46 to over 550^1^ 1Based on fully OA journals listed by Elsevier at https://www.elsevier.com/about/open-science/open-access/open-access-journals on 2016-06-20.(Solomon & Björk, 2012).

As publishing in APC funded OA articles becomes more commonplace there is concern that if libraries begin paying publishing fees in lieu of subscriptions it could become a significant burden for libraries at research intensive universities. A recent survey of libraries found about 20% of the funding for APCs is coming out of library budgets with 70% of the respondents indicating the funding for APCs at their libraries is coming out of the materials budget (Lara, 2014). There is a real concern such a significant shift in funding for scholarly publishing would be unsustainable for research intensive universities in the USA and Canada. The University of California (UC), Davis, is leading a multi-institutional project titled Pay It Forward (PIF) including 4 research intensive universities focused on estimating the likely budgetary impact of such a transition. The research is being funded by the Andrew W. Mellon Foundation (Smith, 2014). As consultants on this project, our major role was in helping estimate the likely cost of APCs for articles published by researchers at research intensive universities in the USA and Canada. This paper summarizes our findings in this area.

There have been several studies that have attempted to characterize the cost of APC funded OA. In 2012 we conducted a comprehensive review of the APC prices for the journals in the Directory of Open Access Journals (DOAJ) that were listed in the directory as charging APCs (Solomon & Björk, 2012). As part of the study we collected the article counts for 2011, the most recent calendar year. We gathered either the listed APC price off the journal web site or our best estimate of the typical APC price when there was not a specific single APC listed. We found that across this broad range of journals the APC prices both raw and weighted by the number of articles published to be around 900 USD.

In 2014 we revisited a subset of the journals included in our previous study. We attempted to limit the sample to those journals which researchers at research intensive universities in the US, Canada and Western Europe would likely publish by selecting only journals from publishers with at least 8 journals of which at least 2 were indexed in the (WoS). We included all APC funded journals from publishers who met the criteria above with less than 30 journals and randomly sampled 30 journals from publishers which published over 30 journals. This resulted in a sample of 187 journals from 9 publishers. We found the number of articles published in these journals increased between 2011 and 2013 by an average 24.5% even when PLOS ONE was left out of the analysis as an outlier. The average APC in this subset of journals was 1,292 USD in the fall of 2012 and had increased to 1,418 USD by the fall of 2014.

Most of the large, traditionally subscription publishers have begun publishing full OA journals We collected APC prices for 102 journals published by 6 major traditionally subscription publishers (Björk & Solomon, 2014). The 102 journals published by major traditionally subscription publishers were on average 679 USD higher than our sample of journals from full OA publishers. Interestingly 15 of the 102 journals from these major publishers had APCs under 500 USD. Many of these journals however were medical journals that only published case reports (Cohen, 2006).

Morrison and colleagues (2015) conducted a study gathering the list price and pricing methodology of the journals in the DOAJ that charge APCs. They used a stratified sampling procedure that selected 1,584 of the 2,567 journals listed in the DOAJ as charging APCs. Their results were similar to our first study finding an average APC of 964 USD suggesting there has been modest inflation in APC prices in the 3 years in between the 2 studies. As with our first study, this study included a wide range of journals many of which authors from research intensive universities in the US, Canada and Western Europe are unlikely to publish.

The previous studies focused on the published prices of full OA journals. Pinfield and his colleagues (2016) conducted a study assessing the total cost to institutions of paying both subscriptions and APCs including APCs from hybrid journals which are subscription journals where authors can pay APCs to make their individual articles OA. They used data from 23 universities in the United Kingdom (UK) gathered between 2007 and the first quarter of 2014. Pinfield and his colleagues also attempted to estimate the administrative costs of paying APCs. They found a significant increase in the total costs to these universities following policy changes in the UK encouraging APC funded OA. By 2013, the APCs paid by these universities to major subscription publishers for hybrid articles increased the total cost of access to these journals by about 10%. They defined the total cost of access as APCs paid for their authors, subscription fees and the administrative costs of paying APCs. They also found it difficult to estimate administration costs and these costs appeared to vary considerably among universities. The APC levels Pinfield and his colleagues found were roughly consistent with our earlier study.

While the studies described above begin to provide a picture of APC pricing and in the case of Pinfield and his colleagues’ study, the total cost of access for of UK universities, we felt we needed additional data that would be more directly applicable for estimating the feasibility of transitioning to APC funded OA for research intensive institutions in the USA and Canada. We believe there is evidence that researchers at research intensive universities tend to publish in the more expensive APC journals and hence previous studies estimating APC prices that included all OA journal would underestimate the cost of APCs for this group of researchers.

The goal of the study was to estimate the per article APC expenditures for the publications of researchers at research intensive universities in the USA and Canada.

## Methods

We used three types of data to characterize what the likely cost of APCs would be for research intensive universities in the USA and Canada. Each has its strengths and limitations. By triangulating different sources of information we felt we could derive a more robust estimate of the likely cost of APCs for researchers at these institutions. Firstly, we tied published APC prices to OA articles published by faculty at the PIF partner universities. Secondly we identified a sample of journals published by major traditionally subscription publishers that were recently converted from subscription to APC funded OA. Thirdly we gathered APC payments made by funding agencies and universities from special budgets set aside for this purpose such as was used by Pinfield and his colleagues in their study of UK university APC funding programs.

**WoS Metadata**—Thomson Reuters in partnership with the PIF Project provided article level metadata from the WoS for the articles published by the PIF partner universities between 2009 and 2013. They also provided article level metadata for the APC payment records we obtained from the universities and funding agencies described below that were matched via Digital Object Identifiers (DOIs). The metadata contained a variety of useful information however for the purposes this analysis we focused on the type of publication limiting the analysis to research articles and conference proceedings. We also broke down the payment and APC pricing results by discipline. The PIF project settled on a 13 category discipline coding scheme derived from Thomson Reuters Essential Science Indicators (ESI) and Scopus’s 23 category discipline scheme. Since there is no ESI code for the arts and humanities, we coded articles and proceedings listed in the Arts & Humanities Citation Index (AHCI) as being in the arts and humanities. The coding scheme developed by the project is shown in Table 3.

**APC Prices for Publications by PIF Partner University Faculty**—As noted earlier, to get an accurate picture of the cost of transitioning from subscription to APC funded OA, we needed to characterize the APC prices for the types of OA journals researchers at research intensive universities in the USA and Canada would likely publish. We matched the articles and proceedings researchers at the 4 PIF partner institutions published between 2009 and 2013 obtained from the WoS with APC pricing data obtained from journal websites collected in 2014 by Morrison and her colleagues. Their pricing data was the most up-to-date and comprehensive data we were able to locate and they had made it available in a public archive. We used International Standard Serial Numbers (ISSN) to match the individual articles or proceedings identified in the WoS with the APC prices for the journals in which they were published.

**Subscription journals converting to APCs**—We attempted to identify journals from major publishers that traditionally published subscription journals which had transitioned from subscription to APC funded OA. To identify these journals, we searched the websites of 7 large publishers as well as the Internet for press releases, blog entries and other indications journals from these publishers flipped from subscription to OA. Once we identified that a journal had “flipped” to OA, we gathered the APC and other metadata from the journal web site.

**APC payment repositories**—We were able to obtain APC payment data from 4 sources. All were based in Europe. These included UK universities; German universities and the Max Plank Digital Library; the Austrian Science Fund (FWF) and the Wellcome Trust. The data were downloaded around March 1, 2015. The specific information provided, requirements, rules for payment, time period in which the payments were made and the currency differed among these data sets. Each is described in more detail below:

*United Kingdom (UK) Universities*—Stuart Lawson and his colleagues at Jisc compiled APC payment data from a number of UK universities (House of Commons, 2014; Lawson, 2014). We combined the two overlapping data sets removing the duplicates. The payments were converted from GBP to USD using an exchange rate of 1.6 which roughly reflected the exchange rate during the period the APC payments were made. The data include both full OA and hybrid payments.

*Wellcome Trust*—The Wellcome Trust maintains a special budget for paying publication charges for the research it funds. The Trust has released APC payments made during their 2012–2013 and 2013–2014 fiscal years (Kiley, 2014; Kiley, 2015). As with the UK university data above, a currency conversion rate of 1.6 was used for converting from GBP to USD. The data include both full OA and hybrid payments.

*German Universities and Foundations*—APC payment records were available for 22 German universities and 5 other participating institutions (Apel et al., 2015). The payment data in EUR were converted to USD using an exchange rate of 1.3 which roughly reflected the exchange rate during the period the payments were made. Payments were only made for publication in fully OA journals.

*Austrian Science Fund (FWF)*—The FWF covers the cost of APCs and other publication charges for researchers they fund. The data for 2013 was available at the time we merged the data with WoS (Reckling & Kenzian, 2014). Unfortunately 2014 data became available just after we requested WoS metadata from Thomson Rueters (Reckling & Rieck, 2015). The data include both full OA and hybrid payments,

The data from these universities and funding agencies was merged with WoS metadata in late April 2015 using DOIs.

## Results

We identified 14,356 OA articles and proceeding published by researchers at PIF partner universities between 2009 and 2013 in OA journals that we were able to obtain APC prices. Please note the article/proceedings were published between 2009 and 2013 while the APC prices were gathered in 2014.

We collected a total of 13,819 payment records from the 4 APC payment databases. A total of 12,172 or 88% were matched with WoS metadata based on DOIs. After removing duplicates, records that were not articles or proceedings or were missing key information, there were 7,629 payment records that were used in the analyses described below.

Table 1 below presents hybrid and full OA payments from the European payment databases and full APC prices for articles and proceedings by authors from PIF universities. The results are broken down by the 13 discipline categories.

**Table 1:** Breakdown of different sources of APC payment/charges by discipline. APCs in US dollars.

	Hybrid payments			Full OA payments			Full OA prices		
Discipline	Payments	*N*	SD	Payments	*N*	SD	Charges	*N*	SD
Arts and humanities	2,168.26	5	1,276.86	*No data*	0	0	1,273.26	19	354.76
Multidisciplinary	2,074.42	16	1,631.24	1,896.48	64	1,355.18	1,345.83	522	50.39
Mathematics	2,579.93	52	908.46	905.60	5	455.97	1,209.79	24	69.60
Clinical Medicine	3,000.33	626	1,082.86	1,870.32	526	584.89	1,753.60	3,456	466.20
Biomedical Research	2,996.56	1,377	1,212.28	1,952.02	1,076	864.70	1,830.36	5,511	552.38
Life Sciences	2,859.62	667	1,164.24	1,876.85	579	716.09	1,789.30	2,286	552.35
Chemistry	2,901.43	370	915.12	2,403.16	47	1,629.68	1,712.00	189	308.93
Physics and Astronomy	2,575.06	241	844.62	1,890.44	190	1,395.89	1,327.90	139	84.72
Engineering	2,718.00	365	903.61	1,669.40	97	737.46	1,900.44	436	453.47
Earth Science	2,905.81	264	824.92	1,523.47	164	706.69	1,599.72	664	331.82
Business and Economics	2,521.58	35	931.65	1,415.65	4	101.74	1,350.00	11	0.00
Psychiatry/Psychology	2,955.87	204	956.31	1,647.01	231	582.40	1,787.35	373	433.94
Social Science	2,736.35	307	878.52	1,822.51	117	407.03	1,940.57	726	460.28
Total	2,886.88	4,529	1,076.15	1,864.53	3,100	838.55	1,775.07	14,356	510.65

The results presented in Table 1 by discipline should be interpreted with caution. There were very few publications in some of disciplines including arts and humanities, mathematics and business/economics. In addition, many of the articles/proceedings coded in disciplines such as the arts, humanities and social sciences are in journals that would generally be considered to be in the biomedical or life sciences. Table 4 presents the number and percentage of articles/proceedings in each journal within each discipline. For example, as can be seen in Table 4, 59% of the articles/proceedings in engineering were published in BMC Bioinformatics and 68% of the article/proceedings in the arts and humanities where published in PLOS ONE. We believe in most cases these article/proceedings describe research that is multidisciplinary but the ESI/AHCI coding scheme we used only assigned a single discipline code to each publication record. For example, one of the articles coded as being in the arts and humanities was titled “Effects of Culture on Musical Pitch Perception” and was published in PLOS ONE.

**Table 2:** Breakdown of different sources of APC payment/charges by discipline. APCs in US Dollars.

Publisher	Mean	*N*	SD
De Gruyter	1,356.00	5	309.46
Elsevier	1,950.00	7	485.63
Nature Publishing Group	5,200.00	1	
Oxford University Press	2,163.33	3	625.81
Springer	1,380.46	13	372.11
Tailor & Francis	1,031.67	3	451.12
Wiley	2,408.00	9	550.63
Total	1,825.20	41	829.68

**Notes.**

APCs reported in US dollars.

We attempted to identify journals from large traditionally subscription publishing houses that have transitioned from subscription to APC funded OA. We were able to locate 41 such journals from 7 major publishers. A summary of the results is presented in Table 2.

With the exception of one outlier, Nature Communications, the APCs charged for these journals appear similar to APCs for APC funded OA journals published by fully OA publishers. Five of these journals, 2 each from Springer and Elsevier and 1 from Oxford University Press were part of the Sponsoring Consortium for Open Access Publishing in Particle Physics (SCOAP3) tendering process (SCOAP^3^, 2015). The APCs for these journals averaged 1,674 USD.

## Discussion

We encountered several challenges in estimating APC prices for journals researchers at research intensive universities in the US and Canada are likely to publish.

• Assigning a single discipline category to each article/proceeding is somewhat artificial given research is often multidisciplinary. While there were two other discipline coding schemes at different levels of specificity in the WoS that assigned multiple discipline codes for each publication, attempting to use a coding scheme with multiple codes per publication to sort out disciplinary differences in APC prices would have been extremely complex and probably not helpful. The current APC market is also concentrated in a few disciplines and there are very few APC funded OA journals that publish in the social sciences and humanities. The articles and proceedings that we found coded in these disciplines were often multidisciplinary and published in journals that generally publish material in the life, medical or biological sciences.
• The APC market is complex and getting even more complex with full OA and hybrid APCs as well as an increasing number of comprehensive “cost of ownership” agreements negotiated between publishers and universities, university consortia and research funders. APC s are often complex with various discounts and fee structures for different types of publications (Björk & Solomon , 2012).
• There is very little information available on the costs associated with paying APCs, both for the publishers and the organizations that are funding APCs nor how these compare with the costs of negotiating and paying subscription fees.
• The current APC market is fluid and subject to market forces brought about by the APC payment policies of the universities, consortia and funding agencies that are increasingly paying APCs as this market evolves (Björk, 2016).

Despite these difficulties we found a pattern in APC list prices and APC payments by universities and funding agencies that were fairly consistent in all 3 sources of data we used in the study.

(1) For researchers at research intensive universities, APCs the paid for the fully OA journals average around 1,800 USD while hybrid journal APCs average about 3,000 USD.
(2) There do not appear to be large discipline differences in APCs. This likely reflects the limitations of the data available. There were very few publications in a number of disciples and those tended to be multidisciplinary.
(3) Based on the very small sample of journals flipped from subscription to OA by major traditionally subscription publishers that publish a major portion of the scholarly literature, the APCs for these journals appear to be similar to journals that were launched as full OA journals.

Our estimates of the APCs for fully OA journals are considerably higher than estimates of APCs in previous research. Our own and Morrison and her colleagues’ estimates of APC charges (Solomon & Björk, 2012; Morrison et al., 2015) reflect the full distribution of OA journals in the DOAJ. Many of those journals with very low APCs are regional journals that researchers at research intensive universities in the USA, Canada and Western Europe are unlikely to publish. In our later study (Björk & Solomon, 2014) we did try to limit the journals included to those which researchers at research intensive universities would likely publish. However, our methodology for achieving this was weak, limiting the sample to journals published by OA publishers with at least 2 journals in the Web of Science. The criteria did result in a significantly higher APC estimate but well below estimates from this study. We feel this is largely due to the methodology used. Our current study used 3 separate approaches. One, probably the most robust, used APC prices for articles and proceedings authored by researchers at the 4 PIF partner universities. The second approach used actual APC payments made by 2 European foundations and the universities in 2 European countries for their researchers’ publications. The third were APC prices for journals “flipped” from subscription to an APC business model by major traditionally subscription publishers. The APC estimates from these three methodologies triangulated at roughly 1,800 USD for articles published in full OA journals. We feel the estimates are probably the best available for APCs that would likely be paid currently for researchers at research intensive universities in the USA, Canada as well as Western Europe.

Many European governments and funding agencies are working towards transitioning all their research publications to OA with a preference for APC funded OA (Schimmer, Geschuhn & Vogler, 2015). The PIF project is modeling a similar transition for research intensive universities in the USA and Canada. At this juncture it appears we are moving towards a wide scale transition of the existing subscription journals to OA publishing much of it funded by APCs (Shearer, 2016). Having reasonable estimates of the likely costs of APCs is essential for modeling the cost of this large scale transition to OA scholarly publishing. The results presented in this paper are based on a number of sources of information and we feel despite their limitations, reflect the best data available for characterizing the per publication APC costs for research intensive universities in the USA, Canada and Western Europe.

## Appendix 1

**Table 3:** Scopus—WOS subject mapping.

Scopus 27	Subject merge	ESI 23
General	Multidisciplinary	E Multidisciplinary (sciences)
S Mathematics	Mathematics	E Mathematics
S Medicine	Clinical Medicine	E Clinical Medicine
S Pharmacology, toxicology and pharmaceutics		E Pharmacology & Toxicology
S Nursing		
S Health professions		
S Dentistry		
S Immunology and microbiology	Biomedical Research Disciplines	E Immunology
		E Microbiology
S Biochemistry, genetics and molecular biology		E Molecular Biology and Genetics
S Neuroscience		E Neuroscience and Behavior
S Agricultural and biological sciences	Life Sciences	E Agricultural sciences
		E Biology and Biochemistry
S Veterinary		E Plant and animal sciences
S Chemistry	Chemistry	E Chemistry
S Chemical engineering		
S Physics and astronomy	Physics and Astronomy	E Physics
		E Space sciences
S Engineering	Engineering	E Engineering
S Materials Science		E Materials Science
S Computer Science		E Computer Science
S Energy		
S Earth and planetary sciences	Earth Sciences	E Geosciences
S Environmental science		E Environment/ecology
S Business management and accounting	Business and economics	E Economics and business
S Decisions sciences		
S Economics, econometrics and Finance		
S Psychology	Psychiatry/Psychology	E Psychiatry/Psychology
S Social Sciences	Social Science	E Social sciences, general
S Arts & Humanities	Arts and humanities	E (Arts and Humanities—category to be created from WOS categories/research areas. PIF team will have to assign journals with both an A&H and SocSci ESI category to a single preferred category)

## Appendix 2

**Table 4:** Number of APC funded publications in each journal within each discipline based on PIF partner authored article/ proceeding 2009–2013. Article Processing Charge (APC) based on Morrison et al. (2015).

Discipline	Journal	APC	Number	Percent
**Arts and Humanities**	ENTROPY	1,349	2	10.5%
	ENVIRONMENTAL RESEARCH LETTERS	1,920	1	5.3%
	PLOS ONE	1,350	13	68.4%
	RELIGIONS	337	2	10.5%
	SCIENTIFIC REPORTS	1,350	1	5.3%
**Multidisciplinary**	DISCRETE DYNAMICS IN NATURE AND SOCIETY	1,200	1	0.2%
	PLOS ONE	1,350	477	91.4%
	SCIENTIFIC REPORTS	1,350	39	7.5%
	SCIENTIFIC WORLD JOURNAL	1,200	1	0.2%
	SYMMETRY-BASEL	562	2	0.4%
	THESCIENTIFICWORLDJOURNAL	1,200	2	0.4%
**Mathematics**	ABSTRACT AND APPLIED ANALYSIS	1,200	2	8.3%
	COMPUTATIONAL AND MATHEMATICAL METHODS IN MEDICINE	1,200	16	66.7%
	FIXED POINT THEORY AND APPLICATIONS	985	1	4.2%
	JOURNAL OF APPLIED MATHEMATICS	1,200	2	8.3%
	PLOS ONE	1,350	3	12.5%
**Clinical Medicine**	AFRICAN JOURNAL OF PHARMACY AND PHARMACOLOGY	600	2	0.1%
	ANNALS OF INTENSIVE CARE	1,930	8	0.2%
	BIOLOGY OF SEX DIFFERENCES	2,285	2	0.1%
	BMC ANESTHESIOLOGY	2,215	5	0.1%
	BMC CANCER	2,215	136	3.9%
	BMC CARDIOVASCULAR DISORDERS	2,215	21	0.6%
	BMC COMPLEMENTARY AND ALTERNATIVE MEDICINE	2,215	23	0.7%
	BMC ENDOCRINE DISORDERS	2,215	11	0.3%
	BMC FAMILY PRACTICE	2,215	12	0.3%
	BMC GASTROENTEROLOGY	2,215	23	0.7%
	BMC GERIATRICS	2,215	17	0.5%
	BMC HEALTH SERVICES RESEARCH	2,215	116	3.4%
	BMC MEDICAL IMAGING	2,215	3	0.1%
	BMC MEDICAL INFORMATICS AND DECISION MAKING	2,215	53	1.5%
	BMC MEDICAL RESEARCH METHODOLOGY	2,215	29	0.8%
	BMC MEDICINE	2,650	54	1.6%
	BMC MUSCULOSKELETAL DISORDERS	2,215	49	1.4%
	BMC NEPHROLOGY	2,215	31	0.9%
	BMC OPHTHALMOLOGY	2,215	9	0.3%
	BMC ORAL HEALTH	2,215	6	0.2%
	BMC PEDIATRICS	2,215	36	1.0%
	BMC PREGNANCY AND CHILDBIRTH	2,215	41	1.2%
	BMC PULMONARY MEDICINE	2,215	13	0.4%
	BMC SURGERY	2,215	2	0.1%
	BMC UROLOGY	2,215	5	0.1%
	BMC WOMENS HEALTH	2,215	10	0.3%
	CANCER MEDICINE	2,250	14	0.4%
	CARDIOVASCULAR DIABETOLOGY	2,185	12	0.3%
	CARDIOVASCULAR ULTRASOUND	1,960	4	0.1%
	CLINICAL EPIGENETICS	2,545	1	0.0%
	CLINICAL INTERVENTIONS IN AGING	2,200	13	0.4%
	DIABETOLOGY & METABOLIC SYNDROME	2,215	3	0.1%
	DIAGNOSTIC PATHOLOGY	2,215	9	0.3%
	DISEASE MARKERS	1,500	13	0.4%
	EVIDENCE-BASED COMPLEMENTARY AND ALTERNATIVE MEDICINE	2,000	68	2.0%
	FRONTIERS IN PHARMACOLOGY	2,194	24	0.7%
	GASTROENTEROLOGY RESEARCH AND PRACTICE	1,500	20	0.6%
	GUT PATHOGENS	2,250	4	0.1%
	HEAD & FACE MEDICINE	2,215	1	0.0%
	HEALTH AND QUALITY OF LIFE OUTCOMES	2,215	33	1.0%
	INFECTIOUS AGENTS AND CANCER	1,960	2	0.1%
	INTERNATIONAL JOURNAL OF CHRONIC OBSTRUCTIVE PULMONARY DISEASE	1,865	12	0.3%
	INTERNATIONAL JOURNAL OF ENDOCRINOLOGY	1,500	11	0.3%
	ITALIAN JOURNAL OF PEDIATRICS	1,960	2	0.1%
	JOURNAL OF CARDIOTHORACIC SURGERY	2,250	25	0.7%
	JOURNAL OF CARDIOVASCULAR MAGNETIC RESONANCE	1,960	41	1.2%
	JOURNAL OF DIABETES RESEARCH	1,500	3	0.1%
	JOURNAL OF ETHNOBIOLOGY AND ETHNOMEDICINE	1,960	3	0.1%
	JOURNAL OF EXPERIMENTAL & CLINICAL CANCER RESEARCH	2,075	11	0.3%
	JOURNAL OF FOOT AND ANKLE RESEARCH	1,960	2	0.1%
	JOURNAL OF HEMATOLOGY & ONCOLOGY	2,250	21	0.6%
	JOURNAL OF OPHTHALMOLOGY	1,500	14	0.4%
	JOURNAL OF ORTHOPAEDIC SURGERY AND RESEARCH	2,545	9	0.3%
	JOURNAL OF OTOLARYNGOLOGY-HEAD & NECK SURGERY	1,960	30	0.9%
	JOURNAL OF TRANSLATIONAL MEDICINE	2,215	82	2.4%
	MALARIA JOURNAL	2,140	124	3.6%
	MARINE DRUGS	2,023	12	0.3%
	MULTIDISCIPLINARY RESPIRATORY MEDICINE	1,960	1	0.0%
	NUTRITION & DIABETES	3,300	14	0.4%
	ONCOTARGETS AND THERAPY	2,200	5	0.1%
	ORPHANET JOURNAL OF RARE DISEASES	2,450	24	0.7%
	PAKISTAN JOURNAL OF MEDICAL SCIENCES	71	2	0.1%
	PARTICLE AND FIBRE TOXICOLOGY	1,960	15	0.4%
	PATIENT PREFERENCE AND ADHERENCE	2,200	28	0.8%
	PEDIATRIC RHEUMATOLOGY	1,960	16	0.5%
	PLOS MEDICINE	2,900	64	1.9%
	PLOS ONE	1,350	1,725	49.9%
	RADIATION ONCOLOGY	1,960	26	0.8%
	REPRODUCTIVE HEALTH	2,215	5	0.1%
	RESPIRATORY RESEARCH	2,625	27	0.8%
	SCANDINAVIAN JOURNAL OF TRAUMA RESUSCITATION & EMERGENCY MEDICI	2,150	2	0.1%
	SCIENTIFIC REPORTS	1,350	31	0.9%
	SCIENTIFIC WORLD JOURNAL	1,200	12	0.3%
	THERANOSTICS	1,168	15	0.4%
	THERAPEUTICS AND CLINICAL RISK MANAGEMENT	2,200	6	0.2%
	THESCIENTIFICWORLDJOURNAL	1,200	6	0.2%
	TOXINS	1,124	7	0.2%
	TRIALS	1,960	53	1.5%
	WORLD JOURNAL OF EMERGENCY SURGERY	1,960	11	0.3%
	WORLD JOURNAL OF SURGICAL ONCOLOGY	2,250	21	0.6%
**Biomedical Research Disciplines**	AIDS RESEARCH AND THERAPY	2,165	23	0.4%
	ALGORITHMS FOR MOLECULAR BIOLOGY	1,960	12	0.2%
	ALLERGY ASTHMA AND CLINICAL IMMUNOLOGY	1,960	9	0.2%
	BEHAVIORAL AND BRAIN FUNCTIONS	2,215	13	0.2%
	BIOMEDICAL ENGINEERING ONLINE	2,215	17	0.3%
	BMC CELL BIOLOGY	2,215	15	0.3%
	BMC DEVELOPMENTAL BIOLOGY	2,215	23	0.4%
	BMC GENETICS	2,215	31	0.6%
	BMC GENOMICS	2,215	354	6.4%
	BMC IMMUNOLOGY	2,215	19	0.3%
	BMC INFECTIOUS DISEASES	2,215	66	1.2%
	BMC MEDICAL GENETICS	2,215	60	1.1%
	BMC MEDICAL GENOMICS	2,215	47	0.9%
	BMC MICROBIOLOGY	2,215	60	1.1%
	BMC MOLECULAR BIOLOGY	2,215	13	0.2%
	BMC NEUROLOGY	2,215	42	0.8%
	BMC NEUROSCIENCE	2,215	46	0.8%
	BRAIN AND BEHAVIOR	2,500	18	0.3%
	CANCER CELL INTERNATIONAL	2,125	9	0.2%
	CELL COMMUNICATION AND SIGNALING	2,500	7	0.1%
	CELL DIVISION	2,125	8	0.1%
	COMPUTATIONAL INTELLIGENCE AND NEUROSCIENCE	1,000	4	0.1%
	EPIGENETICS & CHROMATIN	2,545	16	0.3%
	EVODEVO	2,545	6	0.1%
	FRONTIERS IN AGING NEUROSCIENCE	2,194	15	0.3%
	FRONTIERS IN BEHAVIORAL NEUROSCIENCE	2,194	28	0.5%
	FRONTIERS IN CELLULAR NEUROSCIENCE	2,194	21	0.4%
	FRONTIERS IN COMPUTATIONAL NEUROSCIENCE	2,194	30	0.5%
	FRONTIERS IN HUMAN NEUROSCIENCE	2,194	154	2.8%
	FRONTIERS IN MICROBIOLOGY	2,194	94	1.7%
	FRONTIERS IN MOLECULAR NEUROSCIENCE	2,194	19	0.3%
	FRONTIERS IN NEURAL CIRCUITS	2,194	38	0.7%
	FRONTIERS IN NEUROANATOMY	2,194	16	0.3%
	FRONTIERS IN NEUROINFORMATICS	2,194	16	0.3%
	G3-GENES GENOMES GENETICS	1,950	68	1.2%
	GENES	562	8	0.1%
	IMMUNITY & AGEING	1,960	4	0.1%
	JOURNAL OF CELLULAR AND MOLECULAR MEDICINE	2,500	8	0.1%
	JOURNAL OF INFLAMMATION-LONDON	2,085	10	0.2%
	JOURNAL OF NEUROENGINEERING AND REHABILITATION	2,215	29	0.5%
	JOURNAL OF NEUROINFLAMMATION	2,285	46	0.8%
	MBIO	3,000	121	2.2%
	MEDIATORS OF INFLAMMATION	1,500	21	0.4%
	MOBILE DNA	2,545	6	0.1%
	MOLECULAR AUTISM	2,545	18	0.3%
	MOLECULAR BRAIN	2,000	15	0.3%
	MOLECULAR CANCER	2,215	54	1.0%
	MOLECULAR CYTOGENETICS	1,960	8	0.1%
	MOLECULAR NEURODEGENERATION	2,420	47	0.9%
	MOLECULAR PAIN	2,625	26	0.5%
	MOLECULAR SYSTEMS BIOLOGY	4,114	85	1.5%
	NEURAL DEVELOPMENT	2,545	31	0.6%
	NEUROPSYCHIATRIC DISEASE AND TREATMENT	2,200	25	0.5%
	NEUROSIGNALS	1,798	4	0.1%
	OXIDATIVE MEDICINE AND CELLULAR LONGEVITY	1,500	10	0.2%
	PARASITES & VECTORS	2,015	28	0.5%
	PLOS GENETICS	2,250	322	5.8%
	PLOS NEGLECTED TROPICAL DISEASES	2,250	64	1.2%
	PLOS ONE	1,350	2,639	47.9%
	PLOS PATHOGENS	2,250	242	4.4%
	RETROVIROLOGY	2,215	65	1.2%
	SCIENTIFIC REPORTS	1,350	53	1.0%
	SCIENTIFIC WORLD JOURNAL	1,200	6	0.1%
	THESCIENTIFICWORLDJOURNAL	1,200	8	0.1%
	VIROLOGY JOURNAL	2,215	55	1.0%
	VIRUSES-BASEL	1,573	36	0.7%
**Life Sciences**	BIODATA MINING	1,960	1	0.0%
	BIOLOGICAL PROCEDURES ONLINE	2,250	2	0.1%
	BIOLOGY DIRECT	2,215	12	0.5%
	BIOMED RESEARCH INTERNATIONAL	1,500	38	1.7%
	BIOTECHNOLOGY FOR BIOFUELS	2,545	22	1.0%
	BMC BIOCHEMISTRY	2,215	13	0.6%
	BMC BIOLOGY	2,650	36	1.6%
	BMC BIOPHYSICS	2,215	4	0.2%
	BMC BIOTECHNOLOGY	2,215	20	0.9%
	BMC EVOLUTIONARY BIOLOGY	2,215	124	5.4%
	BMC PLANT BIOLOGY	2,215	61	2.7%
	BMC STRUCTURAL BIOLOGY	2,215	8	0.3%
	BMC SYSTEMS BIOLOGY	2,215	100	4.4%
	BMC VETERINARY RESEARCH	2,215	13	0.6%
	CELL AND BIOSCIENCE	2,015	8	0.3%
	ELECTRONIC JOURNAL OF BIOTECHNOLOGY	1,100	2	0.1%
	EUROPEAN JOURNAL OF HISTOCHEMISTRY	1,028	2	0.1%
	FOOD & NUTRITION RESEARCH	1,645	1	0.0%
	FORESTS	899	13	0.6%
	FRONTIERS IN PHYSIOLOGY	2,194	71	3.1%
	FRONTIERS IN PLANT SCIENCE	2,194	59	2.6%
	FRONTIERS IN ZOOLOGY	2,385	7	0.3%
	GENETICS SELECTION EVOLUTION	1,755	3	0.1%
	INTERNATIONAL JOURNAL OF BEHAVIORAL NUTRITION AND PHYSICAL ACTI	2,500	38	1.7%
	JOURNAL OF BIOLOGICAL ENGINEERING	2,040	11	0.5%
	JOURNAL OF BIOMEDICAL SEMANTICS	1,960	8	0.3%
	JOURNAL OF NANOBIOTECHNOLOGY	2,215	1	0.0%
	JOURNAL OF OVARIAN RESEARCH	1,960	6	0.3%
	JOURNAL OF PHYSIOLOGICAL ANTHROPOLOGY	1,170	1	0.0%
	JOURNAL OF RADIATION RESEARCH	1,371	6	0.3%
	JOURNAL OF THE INTERNATIONAL SOCIETY OF SPORTS NUTRITION	2,215	6	0.3%
	LIPIDS IN HEALTH AND DISEASE	2,215	19	0.8%
	MICROBIAL CELL FACTORIES	1,960	19	0.8%
	NUTRIENTS	1,349	27	1.2%
	NUTRITION & METABOLISM	2,060	16	0.7%
	NUTRITION JOURNAL	2,385	36	1.6%
	ONCOGENESIS	3,300	5	0.2%
	PLANT METHODS	1,990	12	0.5%
	PLOS BIOLOGY	2,900	118	5.2%
	PLOS COMPUTATIONAL BIOLOGY	2,250	194	8.5%
	PLOS ONE	1,350	1,004	43.9%
	PROTEOME SCIENCE	2,215	12	0.5%
	REDOX BIOLOGY	1,500	4	0.2%
	REPRODUCTIVE BIOLOGY AND ENDOCRINOLOGY	2,060	25	1.1%
	SCIENTIFIC REPORTS	1,350	29	1.3%
	SCIENTIFIC WORLD JOURNAL	1,200	2	0.1%
	SYMMETRY-BASEL	562	1	0.0%
	VETERINARY RESEARCH	1,755	12	0.5%
	ZOOKEYS	411	54	2.4%
**Chemistry**	INTERNATIONAL JOURNAL OF MOLECULAR SCIENCES	1,798	47	24.9%
	INTERNATIONAL JOURNAL OF POLYMER SCIENCE	1,200	2	1.1%
	MOLECULES	2,023	28	14.8%
	PLOS ONE	1,350	44	23.3%
	POLYMERS	1,349	6	3.2%
	SCIENTIFIC REPORTS	1,350	17	9.0%
	SENSORS	2,023	44	23.3%
	SYMMETRY-BASEL	562	1	0.5%
**Physics and Astronomy**	ADVANCES IN ASTRONOMY	1,000	7	5.0%
	ADVANCES IN CONDENSED MATTER PHYSICS	1,200	1	0.7%
	ADVANCES IN MATHEMATICAL PHYSICS	1,200	2	1.4%
	ENTROPY	1,349	17	12.2%
	INTERNATIONAL JOURNAL OF PHOTOENERGY	1,200	5	3.6%
	NANOSCALE RESEARCH LETTERS	1,385	17	12.2%
	PLOS ONE	1,350	34	24.5%
	SCIENTIFIC REPORTS	1,350	56	40.3%
**Engineering**	ADVANCES IN ELECTRICAL AND COMPUTER ENGINEERING	274	1	0.2%
	ADVANCES IN MATERIALS SCIENCE AND ENGINEERING	1,200	3	0.7%
	ADVANCES IN MECHANICAL ENGINEERING	1,500	5	1.1%
	APPLIED SCIENCES-BASEL	562	1	0.2%
	BMC BIOINFORMATICS	2,215	259	59.4%
	CRYSTALS	562	3	0.7%
	ENERGIES	1,349	11	2.5%
	EURASIP JOURNAL ON ADVANCES IN SIGNAL PROCESSING	1,455	8	1.8%
	EURASIP JOURNAL ON IMAGE AND VIDEO PROCESSING	1,145	2	0.5%
	EVOLUTIONARY BIOINFORMATICS	1,980	6	1.4%
	FRONTIERS IN NEUROROBOTICS	2,194	1	0.2%
	INTERNATIONAL JOURNAL OF ANTENNAS AND PROPAGATION	1,500	2	0.5%
	INTERNATIONAL JOURNAL OF DISTRIBUTED SENSOR NETWORKS	1,500	4	0.9%
	JOURNAL OF NANOMATERIALS	1,200	12	2.8%
	JOURNAL OF SENSORS	1,000	1	0.2%
	MATERIALS	1,573	21	4.8%
	MATHEMATICAL PROBLEMS IN ENGINEERING	1,200	10	2.3%
	METALS	337	1	0.2%
	MICROMACHINES	562	3	0.7%
	NANOMATERIALS	337	1	0.2%
	OPTICAL MATERIALS EXPRESS	1,350	11	2.5%
	PLOS ONE	1,350	38	8.7%
	SCIENTIFIC REPORTS	1,350	12	2.8%
	THEORETICAL BIOLOGY AND MEDICAL MODELLING	2,215	20	4.6%
**Earth Science**	ADVANCES IN METEOROLOGY	1,200	6	0.9%
	ATMOSPHERE	562	4	0.6%
	BMC ECOLOGY	2,215	3	0.5%
	ECOLOGY AND EVOLUTION	1,950	44	6.6%
	ENVIRONMENTAL HEALTH	2,040	92	13.9%
	ENVIRONMENTAL RESEARCH LETTERS	1,920	83	12.5%
	INTERNATIONAL JOURNAL OF ENVIRONMENTAL RESEARCH AND PUBLIC HEAL	1,798	88	13.3%
	MINERALS	337	4	0.6%
	PLOS ONE	1,350	285	42.9%
	REMOTE SENSING	1,349	20	3.0%
	SCIENTIFIC REPORTS	1,350	11	1.7%
	SCIENTIFIC WORLD JOURNAL	1,200	2	0.3%
	SUSTAINABILITY	1,124	14	2.1%
	WATER	1,124	8	1.2%
**Business and Economics**	PLOS ONE	1,350	11	100.0%
**Psychiatry/Psychology**	ANNALS OF GENERAL PSYCHIATRY	2,545	7	1.9%
	BMC PSYCHIATRY	2,215	36	9.7%
	EUROPEAN JOURNAL OF PSYCHOTRAUMATOLOGY	1,303	6	1.6%
	FRONTIERS IN PSYCHOLOGY	2,194	145	38.9%
	INTERNATIONAL JOURNAL OF MENTAL HEALTH SYSTEMS	1,960	3	0.8%
	PLOS ONE	1,350	171	45.8%
	SCIENTIFIC REPORTS	1,350	3	0.8%
	THESCIENTIFICWORLDJOURNAL	1,200	2	0.5%
**Social Science**	BMC INTERNATIONAL HEALTH AND HUMAN RIGHTS	2,215	14	1.9%
	BMC MEDICAL EDUCATION	2,215	30	4.1%
	BMC MEDICAL ETHICS	2,215	5	0.7%
	BMC PALLIATIVE CARE	2,215	5	0.7%
	BMC PUBLIC HEALTH	2,215	235	32.4%
	GLOBALIZATION AND HEALTH	2,215	29	4.0%
	HARM REDUCTION JOURNAL	2,545	42	5.8%
	HEALTH RESEARCH POLICY AND SYSTEMS	1,960	6	0.8%
	HUMAN RESOURCES FOR HEALTH	2,545	16	2.2%
	IMPLEMENTATION SCIENCE	2,300	52	7.2%
	INTERNATIONAL JOURNAL FOR EQUITY IN HEALTH	2,040	22	3.0%
	INTERNATIONAL JOURNAL OF CIRCUMPOLAR HEALTH	686	16	2.2%
	INTERNATIONAL JOURNAL OF HEALTH GEOGRAPHICS	1,960	21	2.9%
	INTERNATIONAL JOURNAL OF QUALITATIVE STUDIES ON HEALTH AND WELL	1,234	1	0.1%
	JOURNAL OF OCCUPATIONAL MEDICINE AND TOXICOLOGY	2,085	3	0.4%
	MEDICAL EDUCATION ONLINE	1,166	10	1.4%
	PLOS ONE	1,350	192	26.4%
	POPULATION HEALTH METRICS	1,960	11	1.5%
	SCIENTIFIC REPORTS	1,350	1	0.1%
	SCIENTIFIC WORLD JOURNAL	1,200	1	0.1%
	SUBSTANCE ABUSE TREATMENT PREVENTION AND POLICY	1,960	13	1.8%
	SYMMETRY-BASEL	562	1	0.1%

##  Supplemental Information

10.7717/peerj.2264/supp-1 Data S1 Raw Data and Documention

## Additional Information and Declarations

### Data Availability

The following information was supplied regarding data availability: The raw data has been supplied as Data S1.
