# Supplementary material for: Article processing charges for open access publication—the situation for research intensive universities in the USA and Canada
Source: PeerJ. 2016 Jul 21;4:e2264. doi: 10.7717/peerj.2264 (PMC4963224; doi:10.7717/peerj.2264)
Supplement: Data S1 [file peerj-04-2264-s001.zip › Figshare data and documentation/Documentation for data used in Article Processing Charges for Open Access Publication.docx]

**Documentation for data used in Article Processing Charges for Open Access Publication – The Situation in 2016”**

**PIF partner faculty publications 2009 - 2013 with APCs**

This file contains a record for each publication authored by at least one Pay It Forward partner university employee between 2009 and 2013 in a full OA journal. The publications were identified through the Web of Science (WoS) then matched with APC pricing information obtained from Heather Morrison and her colleagues. (<http://dataverse.scholarsportal.info/dvn/dv/oaapc>)

Each record includes the ISSN of the journal, DOI if available, discipline code (see Appendix I in the article for mapping to Essential Science Indicators and Scopus 23 coding) and the APC in USD. Codes are listed below.

**Discipline codes**

1 Arts and Humanities

2 Multidisciplinary

3 Mathematics

4 Clinical Medicine

5 Biomedical Research Discipline

6 Life Science

7 Chemistry

8 Physics and Astronomy

9 Engineering

10 Earth Sciences

11 Business and economics

12 Psychiatry/Psychology

13 Social Science

**European university and funding agency payment data**

These data were obtained from 4 sources, United Kingdom (UK) universities, German universities and research agencies, the Wellcome Trust and the Austrian Science Fund (FWF). Specific information about the data sets can be obtained from the references below:

Lawson, S. (2014): RCUK APC data (2013-14). Figshare. <http://dx.doi.org/10.6084/m9.figshare.1180122> Retrieved May 2015.

Lawson, S. (2015): APC data for 25 UK higher education institutions - 2014. Figshare. <http://dx.doi.org/10.6084/m9.figshare.1305596> Retrieved May 2015.

Kiley R. Wellcome Trust APC spend 2012-13: data file. Figshare. <https://figshare.com/articles/Wellcome_Trust_APC_spend_2012_13_data_file/963054> Retrieved May 2015.

Kiley R. Wellcome Trust open access (OA) spend and compliance monitoring: 2013-14. Figshare. <https://figshare.com/articles/Wellcome_Trust_open_access_OA_spend_and_compliance_monitoring_2013_14/1321361> Retrieved May 2015.

Dataset Fees paid by German Research Institutions. GitHub <http://openapc.github.io/openapc-de/> Retrieved May 2015.

Reckling, Falk; Kenzian, Margit (2014): Austrian Science Fund (FWF) Publication Cost Data 2013. figshare <http://dx.doi.org/10.6084/m9.figshare.988754> Retrieved May 2015.

Reckling, F, Rieck K, (2015): Austrian Science Fund (FWF) Publication Cost Data 2014. figshare. <https://dx.doi.org/10.6084/m9.figshare.1378610.v12> Retrieved May 2015

They contain the Digital Object Identifier (DOI), discipline (see coding scheme above) type of payment (full OA/hybrid) APC in USD and the source of the payment records.

**Type of article** 1 Full OA, 2 Hybrid

**Funder Codes**

1 – FWF

2 – German Universities and institutes

3 – UK data from Jisc

4 – Wellcome Trust

**Flipped Journals from Major Publishers**

41 journals from major publishers that transitioned to OA and were retained by the publisher. Data labels in the first row of the sheet should be self-explanatory.
